# Peer Effects on Academic Performance of High School Students in a County-Level Context of Western China: Empirical Evidence from Large-Scale Social Network Survey

**DOI:** 10.3390/bs16030370

**Published:** 2026-03-05

**Authors:** Pengfei Zhang, Haifeng Du, Peibo Zhu, Xiaochen He

**Affiliations:** School of Public Policy and Administration, Xi’an Jiaotong University, Xi’an 710049, China; pf.zhang@stu.xjtu.edu.cn (P.Z.); haifengdu@mail.xjtu.edu.cn (H.D.); zhupb@stu.xjtu.edu.cn (P.Z.)

**Keywords:** academic performance, peer effects, peer relationships, reflection problem, social networks

## Abstract

Peer relationships are closely associated with the academic performance of adolescent students. This paper develops an integrated framework taking the peer main effect as the starting point to systematically incorporate the demonstration effect, the within-class network effect, and the cross-class average effect. Using comprehensive network and survey data from high school students in a typical county in western China, this paper employs a network-based identification strategy to reveal robust positive peer effects. The mechanism test shows that the demonstration effect can play a moderating role in peer effects on academic performance, with the network effect being heterogeneous across students and the average effect indicative of a potential role in providing diverse academic information. These findings provide empirical insights into the multifaceted nature of peer dynamics, offering actionable evidence for designing targeted interventions to improve educational outcomes in underdeveloped regions.

## 1. Introduction

For a considerable period of time, the study of peer effects on academic performance has received widespread attention in the academic community. Related studies have shown that in the social environment of schools (Rodgers & Rose, 2001; Yekaterina & Leesch, 2023), students’ learning behavior and scores are often influenced by peers (Hanushek et al., 2003; Kang, 2007; Wang et al., 2021; Jiang, 2023; Rehman et al., 2024), especially in classes or social networks with strong interaction and collective consciousness (Rubin et al., 2008; Ezzarrouki, 2016). However, despite this apparent consensus, robustly identifying such effects remains a subject of intense debate due to the reflection problem and selection bias (Manski, 1993; Sacerdote, 2011). Without disentangling genuine influence from homophily and simultaneous interactions, it is difficult to ascertain the true impact of peers on academic outcomes. This scientific clarity is crucial because improving students’ performance not only reflects short-term cognitive abilities (Peng & Kievit, 2020) but also predicts future economic returns (Hout, 2012; French et al., 2014). This is particularly important for high school students who are about to enter adulthood.

In China, the high school stage is the period when students face the most concentrated and intense academic pressure (Sun et al., 2012), and it is also a crucial stage for personal cognitive development and the enhancement of socialization skills (McClintock, 2017). The distinctive class-based teaching model and the prevalent residential system in China inevitably make peers play an important role in students’ academic performance (Ding & Lehrer, 2007; Carman & Zhang, 2012). However, the intense academic competition environment of the college entrance examination often leads students to overlook the positive impact that high-quality peers can have on their academic performance (Stewart, 2007; Gallardo et al., 2016; Walsham et al., 2023), which is more evident in the economically underdeveloped and educationally scarce western regions of China. The western China context provides a unique setting to test the boundary conditions of peer influence. In the vast western regions of China, most high schools are boarding schools, and many students’ parents work away from home (Bai et al., 2017). This means that high school students here, in addition to facing relatively tight educational resources, also encounter a special situation of insufficient family support (Han et al., 2017). Theoretically, this parental absence and boarding environment may increase students’ psychological and functional reliance on peers, potentially amplifying peer effects as a compensatory mechanism for the lack of familial academic socialization. The intense academic pressure often leads students to spontaneously seek emotional support through peer relationships (Stanton-Salazar & Spina, 2005; Chen, 2019; Oduwaye et al., 2023). Nevertheless, they lack the conscious motivation to actively establish high-quality peer relationships in terms of academic performance, neglecting the positive impact that high-quality peer interaction might have on academic performance (Yang & Wang, 2023). To some extent, this exacerbates the imbalance in academic performance and indirectly affects their future well-being (Reschly et al., 2008; Song, 2024). Therefore, it is particularly important to focus the research on the high school student group in the western regions of China and explore the impact of peer effects on their academic performance within the macroscopic field of schools.

Random assignment of peers in natural and quasi-natural experiments is widely regarded as the ideal approach for identifying peer effects (Sacerdote, 2001; Zimmerman, 2003; Carrell et al., 2009), as it aims to obtain objective causal estimates (Mouw, 2006). However, such designs are often costly and limited in duration, which not only makes it difficult to capture long-term, structurally embedded mechanisms but also potentially limits ecological validity by disrupting naturally formed social ties (Kihlstrom, 2021). To bridge this gap, social network analysis serves as a valuable alternative (Luria & Kalish, 2013), preserving authentic relational structures while compensating for the lack of random assignment through advanced identification strategies (Berthelon et al., 2019). Existing studies have examined how students’ centrality in networks (Calvó-Armengol et al., 2009), network density and clustering (Zhou et al., 2023), overall structural configurations (Berthelon et al., 2019), and tie strength (Dokuka et al., 2020) shape academic outcomes. These contributions have substantially enriched our understanding of peer effects from structural and relational perspectives.

Nevertheless, two important gaps remain. First, relatively little is known about how peer academic performance operates within naturally formed high school networks, particularly under conditions of intense academic competition. Second, while prior studies emphasize structural characteristics, insufficient attention has been paid to how peer quality and diverse channels of peer interaction jointly condition the magnitude and mechanisms of peer influence. These gaps are especially salient in the context of high schools in western China, where academic pressure is concentrated and peer interactions are highly structured. Importantly, unlike prior studies that focus solely on network structure or individual attributes, our study integrates both dimensions, constructing measures that capture the combined influence of peers’ structural positions and their academic performance. This structural-attribute integration represents a key conceptual and theoretical contribution of our work. Against this backdrop, this study seeks to address the following research questions:

RQ1: Does peer academic performance significantly impact individual academic achievement among high school students?

RQ2: How does the demonstration effect, characterized by peer quality and the probability of being influenced, moderate peer effects?

RQ3: How do within-class network structures and academic attributes jointly condition the magnitude of peer effects?

RQ4: How does cross-class exposure, through the interaction of network ties and academic attributes, further moderate peer effects?

To answer these questions, we conduct an in-depth investigation of peer effects on academic performance using data from 3847 high school students across 86 classes in two schools, each covering three grade levels, located in a typical county in western China. Building on the research questions outlined above, this study makes several contributions. First, at the theoretical level, we advance the conceptualization of peer influence by developing an integrated framework that captures its multi-dimensional pathways. Moving beyond a simplified view of peer effects, we articulate how academic influence is transmitted through the main effect and the demonstration effect, while explicitly decomposing potential moderation channels within and across classes. By synthesizing these mechanisms, we offer a more holistic theoretical perspective on how individual interactions shape academic trajectories. Second, by constructing core variables based on near-complete peer network data, we provide rare empirical evidence on peer effects among high school students at the county level in western China, thereby enriching the contextual understanding of peer dynamics under conditions of intensive academic competition. Third, to address potential endogeneity concerns, we adopt a layered identification design. Beyond incorporating rich individual controls, school-by-grade fixed effects, and class-level random intercepts in the baseline specification, we construct instrumental variables based on second-order peer performance, including lagged exam scores, self-evaluated peer scores, and homeroom teacher evaluations matched via long-format data, and implement the IV strategy using both two-stage least squares (2SLS). In addition, we perform placebo tests, implement the control function approach, and conduct a series of other robustness checks, all of which consistently support our findings and ensure the credibility and generalizability of the results.

## 2. Theory and Hypotheses

Peer networks are social structures formed by individuals through social interactions with their age-peers or groups (Sweet & Zheng, 2018; Littlecott et al., 2022), which not only influence students’ social behaviors but also have direct modeling effects or subtle indirect influences on their academic performance (Montgomery et al., 2020; Bond et al., 2017; Wu et al., 2023). To clarify these influences, our theoretical analysis distinguishes among four defined pathways: first, the association between peer and individual academic performance; second, the demonstration effect manifested through the moderating role of peer quality; third, the moderation from the within-class interaction between peer network characteristics and peer attributes; and fourth, the moderation from the corresponding cross-class interaction. In China, owing to the substantial pressure of the college entrance examination, students’ daily activity spaces are primarily concentrated in the school. Their social interactions with peers also mainly take place here. Hence, the social relationships that students establish at the school level almost constitute the entirety of their peer networks (Lintner et al., 2024). This is even more so in boarding high schools in the western regions of China. By leveraging this near-complete network structure, we systematically link these specific dimensions to our hypotheses, examining how peer effects are both manifested and moderated within the macroscopic school field.

Our overall view is that the peer effect on academic performance is widely present among high school students in a county-level context of western China, that is, the average academic performance of a student’s peers is systematically associated with the student’s own academic performance (DeLay et al., 2016). Rather than conceptualizing this relationship as a mere statistical association, we frame it as an endogenous peer effect arising from individuals’ embeddedness in naturally formed peer networks. The overall characteristics of the group will shape norms and expectations and have a profound impact on individual behavior through the process of socialization (Harris, 2013). In this sense, peer academic performance constitutes a normative reference environment that structures students’ expectations and behavioral orientations. Since each student is embedded in the peer network, their academic performance is inevitably directly affected by their own characteristics and the endogenous influence of their peers’ academic performance in the network (Yeung & Nguyen-Hoang, 2015). Accordingly, we treat peer academic performance as an endogenous contextual factor within the network, whose aggregate level systematically shapes individual academic outcomes. Therefore, we put forward the following core hypothesis as the main effect hypothesis of our theoretical analysis framework:

**H1.**  *Peer academic performance is positively associated with individual academic performance.*

In terms of specific influence mechanisms, the foremost factor is the demonstration effect of high-achieving peers on academic performance. According to social learning theory (Bandura & Walters, 1977), students are profoundly influenced by their peers during daily interactions, particularly those with higher academic proficiency. This theoretical perspective suggests that high-achieving peers can stimulate an individual’s learning motivation through behavioral modeling, resource sharing, or the application of competitive pressure, prompting students to imitate the learning behaviors of those with superior academic performance, thereby enhancing their own academic outcomes (Moore et al., 2015). This imitation process often begins with psychological benchmarking: from the standpoint of social comparison theory (Buunk & Gibbons, 2007), students tend to compare their performance with that of surrounding peers (Wehrens et al., 2010), and when they perceive substantial disparities relative to higher-performing peers (Dijkstra et al., 2008), such comparisons trigger a psychological convergence response, which in turn transforms into an internal drive to improve their own performance (Lalot & Houston, 2025).

The efficacy of the learning and imitation processes induced by social comparison depends largely on the quality of student-centered peer networks (Humlum & Thorsager, 2021). In this context, peer quality is the core element through which the demonstration effect is materialized and reinforced. When a peer network comprises a higher proportion of individuals who outperform the student, it creates a concentrated environment of superior learning habits (Frijters et al., 2019) and establishes deep interactive feedback mechanisms through effective resource sharing and academic support (Berthelon et al., 2019). This density of high-achieving peers provides a constant, high-standard reference point that generally enhances the academic achievement of all network members (Altermatt & Pomerantz, 2005; Kang, 2007). Thus, peer quality serves as a critical environmental moderating variable that amplifies the positive impact of peer performance.

From the perspective of the specific moderation mechanism, the influence of peer quality operates by altering the potential for upward convergence. A higher proportion of superior peers within a network essentially shifts the student’s relative position within their social environment, thereby increasing the statistical probability that they will be reached and driven by the demonstration effect. In other words, a high-quality peer network functions by maximizing the “odds” of being influenced by academic excellence, transforming the demonstration effect from a latent possibility into a high-probability driver of performance enhancement. Consequently, the strengthening effect of peer quality on individual achievement is realized through this increased likelihood of demonstration-based influence. Based on these internal logics, we further propose:

**H2a.**  *Peer quality positively moderates the relationship between peer academic performance and individual academic performance.*

**H2b.**  *The moderating effect of peer quality operates through an increased probability of demonstration-based influence.*

The demonstration effect proposed in the previous hypothesis is based on the overall moderation mechanism of the student peer network. Since the complete peer network of students can be obtained at the school level, the corresponding moderation mechanism can be further analyzed in more detail in within-class and cross-class contexts based on the students’ class affiliation.

The class network is an overall network composed of all the students in the class and is an important factor influencing students’ academic performance (Mertens et al., 2020; Hill & Peuker, 2023). Based on social capital theory (Nahapiet & Ghoshal, 1998), the class serves as a micro-social system (Keedy, 1995) where peer effects are facilitated through the synergy of structural embeddedness and resource-rich peer attributes. Since students’ daily learning and interaction mainly occur within the class (Mulder et al., 2022), the peer network within the same class and its relative standing in the overall class network significantly shape the intensity of peer influence. From a structural perspective, a within-class peer network located at the center of the class network possesses higher structural social capital. The more a peer network is located at the center, the more connections it has and the broader its influence is. It is more likely to benefit from the information diffusion mechanism in the network and is also more susceptible to the study habits and behavioral patterns of high-achieving students who are not directly connected (Zhang et al., 2016; Vignery, 2022). This structural advantage facilitates the flow of relational social capital, indirectly increasing the possibility for members of the high-centrality peer network to improve their own study behaviors and achieve higher academic performance (Dokuka et al., 2020).

Furthermore, social capital within the class is not only determined by network position but also by the quality of embedded resources, represented by peer attributes. If the overall academic performance of a student’s within-class peers is relatively higher, it signifies a higher stock of cognitive social capital. This will directly magnify the possibility of performance improvement since, at this point, the peer network demonstrates stronger learning capabilities and provides high-intensity, direct academic support through strong ties. This means that the centrality of the student peer network relative to the class network and its academic performance level will have a positive moderation effect on the peer effect on academic performance. Since this moderation effect is achieved by the within-class peer network based on the class network, we call it the network effect of within-class peers. Therefore, we propose the following hypothesis:

**H3.**  *The interaction between within-class peer network characteristics and peer attributes positively moderates the effect of peer academic performance on individual achievement.*

In the cross-class context, as these peers are scattered across different classes, their collective influence cannot be aggregated at a single organizational level; instead, it manifests as a cumulative average of discrete interactions. This paper refers to this as the average effect of cross-class peers. From the perspective of the strength of weak ties theory (Granovetter, 1973), these cross-class relationships function as bridging social capital, providing students with access to non-redundant information and diverse academic perspectives that are unavailable within the dense, redundant circles of their own class. The logic of the potential moderation effect of a single cross-class peer is fundamentally consistent with internal class dynamics, yet it operates through a mechanism of contextual expansion. The potential moderation effect remains contingent upon the peer’s centrality within their own class and their relative academic performance—the former governing their resource acquisition capacity (Trefalt, 2014) and the latter determining their resource conversion efficiency. Unlike the high-intensity, normative pressure exerted by strong-tie peers within the same class, these weak-tie connections facilitate a broader exposure effect, allowing students to internalize a more heterogeneous set of academic norms and innovative learning strategies from diverse social environments. These factors jointly determine the strength of their moderation ability on academic performance. Hence, we propose the following hypothesis:

**H4.**  *The interaction between cross-class peer network characteristics and peer attributes positively moderates the effect of peer academic performance on individual achievement.*

**Figure 1:**
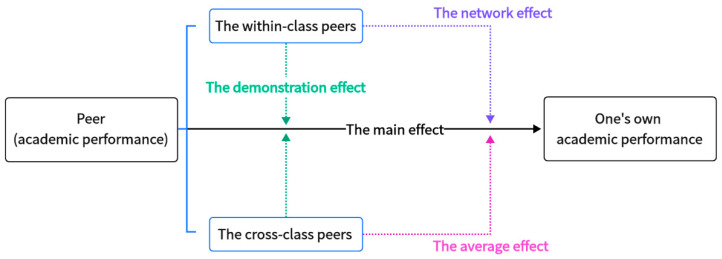
Theoretical analysis framework of peer effects on academic performance.

Based on the foregoing analysis, the theoretical framework of this paper is summarized in Figure 1. This framework provides both theoretical and empirical foundations for studying peer effects on academic performance, encompassing the main effect and the demonstration effect, while explicitly decomposing potential moderation channels within and across classes. Of course, decomposing the moderation effects necessitates technical improvements to the estimation model. A concise introduction to these enhancements is provided in Section 3 of this paper, while more detailed technical specifications can be found in Appendix A.

## 3. Materials and Methods

### 3.1. Data Collection

This paper uses the entire data of high school from the base period questionnaire and social network data of the “West County Children and Youth Development Panel Studies” conducted by the research group of New Urbanization and Sustainable Development of Xi’an Jiaotong University in 2023. This survey was a comprehensive census covering nearly all junior and senior high school students in a typical county in the western regions of China. Only students who declined to participate, were temporarily absent on the day of the survey, or were from a few very remote areas were not included, but their number was negligible. In addition to students, the survey also collected information from homeroom teachers and school administrators. The total sample size of the questionnaires was 11,150, with 5123 nodes and 86,033 edges in the social network data. Social networks were captured at the within-school level, covering six types of relationships: study, friendship, heart-to-heart talk, assistance, play, and negative interactions. The questionnaire collected extremely comprehensive information, including student characteristics, family background, educational experiences, migration and left-behind experiences, as well as community and other relevant contextual factors.

The selection of this specific county is grounded in its typicality as a rural-urban transitional space in inland China. It possesses the common institutional features of the “County High School” system, where high academic intensity, boarding-based management, and relatively constrained educational resources create a specific social environment for adolescents. As such, the student-to-student interaction patterns and the school-centered social structures observed here are representative of the prevailing educational ecology in many county-level administrative divisions across western China. While this census-based approach provides high internal validity, we acknowledge the contextual limitations when generalizing these findings to elite urban schools in eastern China, where social network formation and academic competitive dynamics may follow different institutional logics. These features establish a solid and scientifically grounded data foundation for our research.

In terms of data utilization, our overall approach is to compute all core independent variables, exogenous variables, moderation variables, instrumental variables (except for the instrumental variable in the long-format data), and other variables by utilizing friendship relationships in social network data in conjunction with the questionnaire data. The decision to focus exclusively on friendship is grounded in both theoretical and empirical considerations. First, friendship represents a multiplex relationship among adolescents that typically encompasses other interaction types such as study, heart-to-heart talk, assistance, and play. Because these positive interactions exhibit substantial structural overlap with friendship, the friendship network serves as a comprehensive and reliable proxy for the functional social capital available to students. Second, alternative tie definitions present significant econometric and conceptual challenges: study relationships are inherently endogenous to academic outcomes as students often sort based on performance, while negative interactions do not align with the normative influence mechanisms theorized in this study. Therefore, friendship provides the most conceptually pure definition of a peer, capturing the sustained emotional and social ties that facilitate behavioral modeling and resource sharing. After data matching and cleaning, the final analytical sample consists of 3847 individuals.

### 3.2. Ethics and Consent

The study protocol was approved by the Ethics Committee of the First Affiliated Hospital of Xi’an Jiaotong University. All procedures were conducted in accordance with the Declaration of Helsinki and local education authority regulations. Prior to data collection in late 2023, written informed consent was obtained from all participating students and their legal guardians.

Participants were explicitly informed of the study’s purpose and their right to withdraw at any time without penalty. To ensure the confidentiality of the peer nomination data, all records were anonymized using unique numeric IDs. Paper-based questionnaires are securely stored for a five-year retention period, while electronic data are kept on encrypted servers, accessible only to the authorized research team.

### 3.3. Identification Strategy

The primary objective of this study is to identify the endogenous peer effect within a network-based linear-in-means framework. In such models, identification is challenging because peer outcomes may be correlated with individual outcomes through multiple channels, including correlated effects, selection in network formation, and reflection problem (Manski, 1993). If not properly addressed, these channels can bias estimates of peer effects.

Following Bramoullé et al. (2009), identification becomes feasible when the strict assumption of fully overlapping group interactions is relaxed and the actual social network is observed. In particular, non-transitive network structures, where a friend of a friend is not necessarily a direct friend, generate exogenous variation that can be exploited to separate endogenous peer effects from correlated and contextual effects. This approach is also supported by Yu et al. (2023), who demonstrate that partial network observability allows for identification of endogenous peer influence.

In the present study, the availability of complete within-school social network data provides the structural foundation for this identification strategy. The observed network topology allows us to exploit variation from indirect peer connections while excluding direct reciprocal influence. Moreover, the inclusion of rich individual, family, and peer-level covariates, together with school-by-grade fixed effects and class-level random intercepts, serves to systematically reduce bias arising from correlated effects and network selection. Specifically, school-by-grade fixed effects absorb shared cohort-specific and institutional influences within each school, such as grade-specific examination schedules, competitive intensity, and school-level management practices that may jointly shape peer composition and academic outcomes. Class-level random intercepts further capture unobserved classroom environments, including teacher characteristics and class-specific norms, thereby addressing cross-level dependence within classes. Importantly, the control variables at the individual, family, and peer levels are conceptualized as pre-determined background characteristics rather than mediating channels of peer influence. They account for baseline heterogeneity that may jointly shape friendship formation and academic outcomes. From a causal perspective, these variables block potential backdoor paths without conditioning on downstream outcomes of peer influence, thereby mitigating the risk of post-treatment bias.

To further address simultaneity, we implement instrumental-variable strategies and dyadic long-format data constructions in subsequent analyses. These approaches leverage exogenous variation from indirect peers and satisfy relevance and exclusion assumptions under the maintained network conditions. Following this identification strategy, the endogenous peer effect is estimated using two-stage least squares (2SLS) and the control function approach, with the latter serving as a robustness check to validate the results of the former. This dual-estimation framework ensures the consistency and reliability of our peer-effect estimates, particularly by demonstrating that the findings remain stable across different methodological assumptions. By confirming that both approaches yield coherent results, we can more confidently rule out potential biases stemming from reciprocal causality or unobserved classroom-level shocks.

### 3.4. Regression Model

To estimate peer effects on academic performance, we implement a three-level modeling strategy. We begin with the baseline linear-in-means specification to identify the endogenous influence of peers’ academic performance. We then extend this model by decomposing peer effects into within-class and cross-class components, which serves both within- and cross-class mechanism analyses. Finally, to examine the mechanisms of mediated moderation, we employ the mediated moderation (meMO) framework (Kwan & Chan, 2018).

Our baseline model adheres to Manski (1993)’s interpretation of peer effects, focusing on the endogenous influence of peers’ academic performance while controlling for individual characteristics, contextual peer characteristics, and correlated effects. Under this framework, we specify a network-based linear-in-means model with school-by-grade fixed effects and class-level random intercepts to account for institutional and classroom-level heterogeneity, and the estimation equation is:

(1) $$y_{i}=\alpha +\rho {\bar{y}}_{-i}+\beta {\bar{X}}_{-i}+\gamma X_{i}+\delta_{g(i)}+u_{c(i)}+\varepsilon_{i},$$

where ${\bar{y}}_{-i}$ and ${\bar{X}}_{-i}$ denote the leave-one-out average outcome and characteristics of student i’s peers in the observed social network, $X_{i}$ denotes individual characteristics that control for observable heterogeneity, $\delta_{g(i)}$ captures school-by-grade fixed effects, $u_{c(i)}$ is a class-level random intercept, and $\varepsilon_{i}\sim N(0,\sigma^{2})$ is the individual error term.

To facilitate the mechanism analysis, we decompose the overall peer effect into within- and cross-class components:

(2) $$y_{i}=\alpha +\rho_{\mathrm{cls}}{\bar{y}}_{-i,\mathrm{cls}}^{w}+\rho_{\text{non-cls}}{\bar{y}}_{-i,\text{non-cls}}^{w}+\beta {\bar{X}}_{-i}+\gamma X_{i}+\delta_{g(i)}+u_{c(i)}+\varepsilon_{i},$$

where ${\bar{y}}_{-i,\mathrm{cls}}^{w}$ and ${\bar{y}}_{-i,\text{non-cls}}^{w}$ represent the weighted peer academic performance within-class and across-class, respectively. The relationship between this model and the baseline linear-in-means model, as well as its derivation, is provided in Appendix A.

To examine the mediated moderation mechanisms underlying peer effects, we adopt the following mediated moderation (meMO) specification:

(3) $$DP_{i}=a_{0}+a_{1}PQ_{i}+\epsilon_{DP},$$

(4) $$y_{i}=b_{0}+b_{1}DP_{i}+b_{2}DP_{i}\times {\bar{y}}_{-i}+{c_{1}}^{\prime }{\bar{y}}_{-i}+{c_{2}}^{\prime }PQ_{i}+{c_{3}}^{\prime }PQ_{i}\times {\bar{y}}_{-i}+\epsilon_{y},$$

where $DP_{i}$ denotes the probability of demonstration and $PQ_{i}$ represents peer quality. In this specification, our primary interest lies in the mediated moderation effect $a_{1}b_{2}$ and the total moderation effect ${c_{3}}^{\prime }+a_{1}b_{2}$. The significance of the parameters was tested using the bootstrap method with 1000 resamples.

### 3.5. Estimation Method

We adopt a multilevel linear model to estimate the peer effects on academic performance because students have a clear hierarchical structure, with individuals nested within classes, classes nested within grades and grades nested within schools. Different factors at various levels jointly influence academic performance. To address this issue, we standardized the scores at the school-by-grade level and used a multilevel linear model to control for school-by-grade fixed effects and the random effects at the class level, accurately estimating the peer effects and avoiding the bias caused by within-group correlation. To validate the necessity and appropriateness of this hierarchical approach, we report the Intraclass Correlation Coefficient (ICC) for each model in the Results section. These diagnostics generally show substantial class-level clustering (with many ICC values around 0.5 or higher), confirming that a multilevel specification is required to account for the nested data structure. When using the multilevel linear model, to avoid multicollinearity, we centered all continuous variables except for the binary variables, which is in line with the standard procedure.

For the endogenous problem caused by selection and reflection problem, since the multilevel linear model we used has controlled for the school-by-grade fixed effects (absorbing systematic differences at the school and grade levels), random class intercepts (absorbing differences in class average levels), and a series of individual control variables (reducing the impact of student-level selectivity on the estimation of peer effects), it has mitigated the selection problem to the greatest extent possible. To address the reflection problem, we employ the two-stage least squares (2SLS) estimation, utilizing the characteristics of second-order friends as instrumental variables. To account for the hierarchical nature of our data, we cluster the standard errors at the class level and incorporate school-by-grade fixed effects to net out unobserved correlated effects. Furthermore, as a robustness check for our identification strategy, we apply the control function approach. Following the standard procedure for control function estimation, we use the bootstrap method (1000 resamples) to obtain consistent standard errors, thereby ensuring the reliability and stability of our statistical inferences.

### 3.6. Variable Settings

Dependent variable: academic performance. This paper uses the midterm exam scores of the 2023 autumn semester as a proxy variable for student academic performance. Given that scores across different schools and grades are often not directly comparable, we standardized the scores at the school-by-grade level.

Core independent variables: The core independent variable of this paper is the leave-one-out average score of peers ${\bar{y}}_{-i}$. But formally, we have decomposed it into two core independent variables, namely the weighted average score of within-class and cross-class peers:

(5) $${\bar{y}}_{-i,\mathrm{cls}}^{w}=w_{-i,\mathrm{cls}}\cdot {\bar{y}}_{-i,\mathrm{cls}},$$

(6) $${\bar{y}}_{-i,\text{non-cls}}^{w}=w_{-i,\text{non-cls}}\cdot {\bar{y}}_{-i,\text{non-cls}}.$$

For the relevant details of this decomposition, please refer to Appendix A. In cases where effect decomposition is not required, our core independent variable naturally becomes the sum of the two, that is:

(7) $${\bar{y}}_{-i}={\bar{y}}_{-i,\mathrm{cls}}^{w}+{\bar{y}}_{-i,\text{non-cls}}^{w}.$$

Moderating variables: We define peer quality as the proportion of peers in an individual’s peer network who have higher scores than the individual’s own score:

(8) $$PQ_{i}=\frac{1}{\left|N\left(i\right)\right|}\left|\left\{j\left|j\in N\left(i\right),y_{j}>y_{i}\right.\right\}\right|,$$

where $N\left(i\right)$ denotes the set of peers of i.

The mediator of the moderating effect of peer quality, namely the probability that students are influenced by demonstration, is defined on the basis of the odds ratio. To avoid its divergence, we normalize it to the interval $\left[0,1\right]$:

(9) $$DP_{i}=\frac{\int_{y_{i}}^{\infty }\hat{f}\left(x\right)dx}{\int_{-\infty }^{y_{i}}\hat{f}\left(x\right)dx+1}=\frac{1-F\left(y_{i}\right)}{1+F\left(y_{i}\right)},$$

where $\hat{f}\left(x\right)$ is the probability density function of the distribution of peer scores, which is estimated by kernel density estimation.

The moderating variable $M_{i}^{cls}$ of within-class peers for sample i is defined as:

(10) $$M_{i}^{cls}=BC_{i}^{sub}\times PR_{i}^{sub},$$

where $BC_{i}^{sub}$ is the betweenness centrality of the sub-network centered on sample i to measure the positional significance of the peer network within the class network, and $PR_{i}^{sub}$ measures student i’s average academic score rank among their within-class peer network. The definitions of the two are as follows:

(11) $$BC_{i}^{sub}=\frac{\sum_{u\in V}\sum_{v\in V,v\neq u}\sum_{p\in P_{u\to v}}I\left(p\cap N_{cls}\left(i\right)\neq \emptyset \right)}{\sum_{u\in V}\sum_{v\in V,v\neq u}\left|P_{u\to v}\right|},$$

(12) $$PR_{i}^{sub}=\frac{1}{N_{cls}\left(i\right)}\sum_{j\in N_{cls}\left(i\right)}{\mathrm{rank}}_{j},$$

where V is the vertex set of the class network, $N_{cls}\left(i\right)$ is the set of the student i’s within-class peers, $P_{u\to v}$ is the set of all the shortest paths from the fixed point u to v, ${\mathrm{rank}}_{j}$ is the percentile rank of student j within the class, ranging from 0 to 1, and I is the indicator function.

The moderating variable $M_{i}^{non-cls}$ of cross-class peers for sample i is defined as:

(13) $$M_{i}^{non-cls}=\frac{\sum_{j\in N_{non-cls}\left(i\right)}BC_{j}^{node}\cdot SR_{j}^{node}}{\left|N_{non-cls}\left(i\right)\right|}.$$

where $BC_{j}^{node}$ is the measurement of the betweenness centrality of the node j, $SR_{j}^{node}$ is the academic performance level of sample j relative to its class, and $N_{non-cls}\left(i\right)$ is the set of the student i’s cross-class peers. The definitions of the two are as follows:

(14) $$BC_{j}^{node}=\frac{\sum_{u\in V}\sum_{v\in V,v\neq u}\sum_{p\in P_{u\to v}}I\left(p\cap \{j\}\neq \emptyset \right)}{\sum_{u\in V}\sum_{v\in V,v\neq u}\left|P_{u\to v}\right|},$$

(15) $$SR_{j}^{node}=\frac{y_{j}}{\sum_{v\in V}y_{v}/\left|V\right|},$$

where V is the set of students in the class to which sample j belongs.

Control variables: In this paper, control variables are selected from the individual, family and peer levels respectively. At the individual level, eight variables are selected, such as age, gender, whether they live in school, study effort, educational expectation, self-confidence, academic performance requirements and health. At the family level, eleven control variables were selected, including household registration, family structure, family economic status, parents’ emotional status, number of children, extracurricular education expenditure, school education expenditure, other education expenditure, family education time investment, family collection of books, and whether family members could speak English. At the peer level, four control variables were selected: the number of friends, the degree of study effort of friends, the number of friends with bad behavior, and the number of friends who had been class leaders. Furthermore, we also controlled the exogenous effects of peers through averaging. For the measurement and description of the control variables, please refer to Supplementary Material.

It is important to note that the dependent variable, core independent variables, and moderating variables in this study are all derived from objective administrative records or relational data, which do not involve psychometric scaling. Similarly, the vast majority of control variables represent objective demographic and socioeconomic facts. For the remaining few self-reported indicators, they are treated as observed manifest variables with high face validity rather than multi-item latent constructs; therefore, traditional reliability and validity tests (such as Cronbach’s alpha) are not applicable in this context.

### 3.7. Descriptive Statistics

Table 1 shows the descriptive statistical information of main variables in this paper, except for the fixed effect variables. As mentioned earlier, in order to render the scores of different schools and grades comparable, we standardized the student scores at the school-by-grade level. For individual variables with outliers, we applied a tail-trimming treatment. For a very small subset of observations with missing values due to apparent random recording errors, we applied mean imputation. Given the negligible proportion of missing data and the plausibly Missing Completely at Random (MCAR) mechanism, this treatment is unlikely to materially affect the estimation results. Owing to the space constraint, the descriptive statistics of the exogenous average variables of the peer characteristic variables are not presented.

It should be noted that although we actually use the centralized variables in practice, here we still report the statistical information of the original variables to show more details of the variables.

## 4. Visualization Analysis

**Figure 2:**
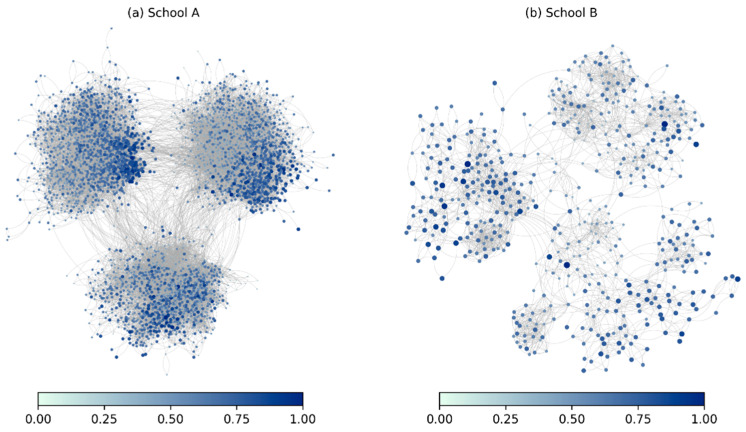
The peer effects on academic performance in the real network topology.

Before the formal regression analysis, we first conducted an analysis of the peer effect on the academic performance of the sample through visualization technology. This paper adopts two visualization approaches. One is based on the actual network topology, while the other describes its distribution. Figure 2 presents the network topologies of two schools, and we have carried out technical processing on them—the larger the node and the darker the color, the better the academic performance. Evidently, there are several hotspots in the figure. High school students with similar academic performances always tend to cluster, which has been verified in the network topologies of both schools.

The network topology can visually reveal the aggregation of students’ academic performance, yet it is not easy to observe the relationship details between a student’s academic performance and that of his or her peers. For this reason, we visualized its distribution. For both standardized scores and unstandardized raw scores, we used the current period’s score and the score from the previous period as proxy variables and carried out the following operations: First, the average scores of peers with higher and lower grades than each sample in their peer networks were calculated. Second, all the sample scores were arranged in ascending order from smallest to largest. Finally, the scores of each sample, the average score of peers whose scores were higher than their own, and the mean score of peers whose scores were lower than their own were plotted.

**Figure 3:**
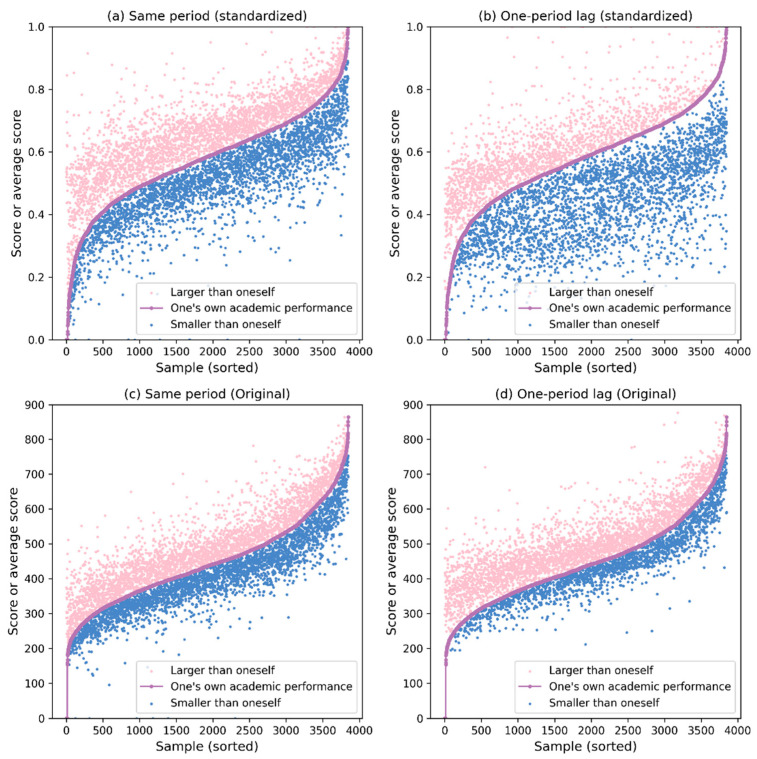
Visualization analysis of peer effects on academic performance.

There are two points that need clarification. One is that the reason why we still use the lagged one-period score as a proxy variable is that we consider the robustness of the visualization analysis results. Since interpersonal relationships have relative stability in a short period of time (Feld, 1997), the possibility of significant changes in the peer network in a short period of time is relatively low. Therefore, using the lagged one-period exam score is an appropriate choice. The second point is that the reason why we perform this operation on both standardized and raw scores is that we can clearly discover the increased variability after standardizing the variables through the standardized score relationship graph. This is crucial for the subsequent regression because it can ensure the objectivity and scientific nature of the regression results. However, the drawback is that it provides less help for the graphical illustration of the peer effect here because standardization reduces the scale of the data and loses the interpretability of the original data. Therefore, we also perform this operation on the raw data to make up for the lost relationship details. The final visualization result is shown in Figure 3.

Figure 3a,b present the visualization results of the standardized scores, while Figure 3c,d show the results of the original scores. Whether standardized or not, we can clearly see that there exists a strong correlation between the academic performance of high school students themselves and that of their peers, that is, students with high academic performance tend to have peers with high academic performance. From the non-standardized results, we can observe that as academic performance improves, high-achieving students are less likely to befriend students with lower academic performance, and vice versa, suggesting a tendency for students to form friendships with peers of similar academic ability.

The visualization results presented above are exploratory and descriptive in nature, while the regression framework provides a formal setting for statistical inference and hypothesis testing. To investigate peer effects within a regression framework, with causal interpretation conditional on the identification assumptions discussed above, we proceed to conduct regression analyses in the next section.

## 5. Results

### 5.1. Analysis of Peer Effects on Academic Performance

At present, we can estimate the peer effects on the academic performance of high school students based on Equation (1), as shown in Table 2. Model (1) is a null model, with an Intraclass Correlation Coefficient (ICC) of 0.565, indicating that the use of a multilevel linear model in this study is reasonable. Model (2) introduces the core independent variable, and the results show that there is indeed a significant positive correlation between the academic performance of peers and that of the students themselves. Models (3), (4), and (5) respectively introduced control variables, exogenous effects, and correlated effects (school-by-grade fixed effects) on the basis of the previous model, all of which significantly confirmed the conclusion of Model (2). Therefore, Hypothesis H1 is verified.

Beyond the average performance of the peer group, the heterogeneity in peer quality may also play a crucial role. Specifically, high-quality peers may exert a moderation effect on the academic transmission process among high school students. Building upon Model (5), we introduced an interaction term between peer academic performance and peer quality. The estimation results reported in Table 3 indicate that the coefficient of the interaction term is statistically significant, suggesting that peer quality moderates the relationship between peers’ performance and individual academic achievement. This pattern is consistent with the hypothesis that the strength of peer effects depends on the overall quality of the social network. Accordingly, Hypothesis H2a is supported by the empirical evidence.

Subsequently, a question emerged for us: How does the quality of peers moderate the peer effect? That is to say, what is the moderation mechanism like? We further introduced the probability of high school students being influenced by their peers and addressed this issue through the meMO model, as specified in Equations (3) and (4). The estimation results are shown in Table 4. Based on the estimation results of Models (7) and (8), we calculated the size of the mediated moderation effect to be 0.461. To test its significance, we employed the Bootstrap method with 1000 resamples to construct bias-corrected confidence intervals. The results indicate that the mediated moderation effect is statistically significant, with a 95% confidence interval of [0.336, 0.601]. This confirms that peer quality moderates the peer effect on academic performance by enhancing the probability of students being influenced by the demonstration effect.

In addition, the direct moderation effect was estimated to be −0.270, with a 95% confidence interval of [−0.410, −0.130], indicating a statistically significant negative effect. The total moderation effect remains positive and significant at 0.190, with a 95% confidence interval of [0.116, 0.278]. These results are consistent with the view that the demonstration effect may play a role in conditioning the transmission of peer academic influences, suggesting that peer quality moderates this relationship. Accordingly, Hypothesis H2b receives empirical support.

It is worth noting that, regarding the temporal ordering in the mediated moderation model, the two components capture distinct analytical dimensions. The quality of the peer group reflects the structural composition of the social environment, which logically precedes and constrains the potential for behavioral influence. In contrast, the probability of students being influenced by demonstration represents the realization of behavioral signals within this environment. Conceptually, the structural peer environment forms the foundation that defines the potential pathways through which peers’ behaviors can be internalized and emulated. This ordering justifies the sequence assumed in the meMO model, with peer quality establishing the context in which the probability of being influenced by demonstration operates, supporting the interpretation that the mediated moderation effect reflects how structural characteristics shape behavioral transmission.

Following the theoretical decomposition of peer moderation into within-class and cross-class components, we utilize Equation (2) to estimate these distinct channels. Within the class, the results of the interaction analysis are presented in Model (9) of Table 5. The estimated coefficient for the interaction term between within-class peer performance and the network effect is −0.084, with a *p*-value of 0.102. This finding contrasts with our initial theoretical expectation, as it is consistent with the possibility that the positive association between peer influence and individual achievement diminishes as the network effect increases. This pattern suggests that the academic benefits of peer spillover may be less pronounced for students with a stronger network position and greater access to class resources.

To further investigate this unexpected mechanism, we calculated the simple slopes of peer influence at three representative levels of the network effect, as presented in Table 6. For students whose within-class network effect is at the lower end, defined as one standard deviation below the mean, the peer influence is at its maximum and is highly significant, with an estimated effect of 0.186 and a *p*-value of less than 0.001. For students at the average level of the network effect, the positive effect remains significant but decreases in magnitude to 0.102, with a *p*-value of less than 0.001. However, for students characterized by a high network effect, defined as one standard deviation above the mean, the peer influence coefficient further declines to 0.018 and fails to reach statistical significance, with a *p*-value of 0.739. Consequently, while Hypothesis H3 is not supported by the empirical evidence, our results reveal the heterogeneous moderating role of the network effect: compared to students with high network effects, those at lower levels of the network effect exhibit stronger marginal effects. This suggests that for these individuals, peer performance serves as a critical external support for acquiring academic information, whereas a high network effect may produce a certain “saturation” effect.

Beyond the analysis of within-class effects, we examined the influence of peers in the cross-class context, as reported in Table 7. The estimated moderation coefficient of 0.225 is positive but not statistically significant, with a *p*-value of 0.354, so it cannot be distinguished from zero. While the sign aligns with theoretical expectations that cross-class interactions may facilitate access to diverse academic resources, the statistical evidence is inconclusive, and no definitive conclusion can be drawn regarding Hypothesis H4. The coefficient nevertheless provides a directional reference for future research.

### 5.2. Endogeneity and Its Treatment

The similarity in academic performance among high school students may not solely arise from socialization-based peer effects. It may also reflect a selection effect (Sacerdote, 2001), whereby students tend to interact with peers who exhibit similar academic performance (Barnes et al., 2014; Vignery, 2021), as well as the reflection problem, in which students simultaneously influence one another. Although randomized peer assignment provides a cleaner identification strategy (Carman & Zhang, 2012; Hasan & Bagde, 2013), such conditions are not available in our heterogeneous sample.

In our baseline and mechanism analyses, rich individual controls together with school-by-grade fixed effects and class-level random intercepts have already been incorporated, which helps mitigate correlated effects and alleviates potential selection bias (Burke & Sass, 2013). However, simultaneity arising from mutual peer influence may still bias the estimation.

Following Bramoullé et al. (2009, 2020), endogenous peer effects can be identified under non-transitive network structures, that is, when a friend of a friend is not necessarily a direct friend. Based on this identification logic, we construct an instrumental variable using the lagged academic performance of second-order peers, consistent with the empirical strategy adopted in Feng (2022). Specifically, for student i, the instrument is defined as:

(16) $$IV_{i}=\frac{\sum_{j\in N(i)}\sum_{\begin{array}{l} k\in N(j)\\ k\notin N(i),k\neq i \end{array}}{\mathrm{score}}_{k}}{\sum_{j\in N(i)}\left|\left\{k\in N(j):k\notin N(i),k\neq i\right\}\right|}.$$

This strategy exploits exogenous variation from higher-order network connections to address the reflection problem. For robustness, we further replace the lagged exam scores with second-order peers’ self-evaluated academic performance and reconstruct the instrumental variable accordingly. It should be noted that the sample size decreases for the two second-order peer instruments, because the second-order peer measures are only defined for students with indirect peer connections.

Table 8 shows the estimated results for the two instrumental variables, model (11) is the result of using lagged academic performance as an instrumental variable, and model (12) is the estimated result using student’s self-evaluated performance as an instrumental variable. Both instrumental variables pass the relevant diagnostic tests. Specifically, the Cragg–Donald Wald F statistic and the heteroskedasticity-robust Kleibergen–Paap rk Wald F statistic exceed conventional thresholds, indicating no evidence of weak instruments, and the Kleibergen–Paap rk LM statistic rejects the null of underidentification. The two instruments yield similar positive estimates, suggesting that the results are consistent across specifications. These findings support the robustness of the analysis and lend confidence to the identification strategy.

Furthermore, we construct an additional instrumental variable by transforming the questionnaire data into long (dyadic) format: for each sample $x_{i}$ in dataset D, we extract each peer’s sample $x_{j}$ from the peer set $N\left(i\right)$ and concatenate $x_{i}$ with $x_{j}$. After concatenation, the dimensionality of the feature space will double, and the final generated long-format dataset is:

(17) $$D_{new}=\overset{n}{\underset{i=1}{\cup }}\underset{j\in N\left(i\right)}{\cup }\left(x_{i},x_{j}\right)$$

where $x_{i}={\left(x_{i1},x_{i2},\ldots ,x_{ik}\right)}^{T}$ and the final sample size is $\sum_{i=1}^{n}\left|N\left(i\right)\right|$. On this basis, for each focal student, we match the corresponding peers with homeroom teachers’ evaluations, covering both cognitive and non-cognitive dimensions, and aggregate them into a composite measure. Since these teacher assessments are correlated with peers’ academic performance but plausibly orthogonal to the focal student’s idiosyncratic error term, they provide an independent source of instrumental variation.

We carried out the regression presented in Table 9 on the matched dataset. Model (13) shows that the results remain effective and consistent with the findings derived from the second-order peer instrument. This alignment, despite variations in effect magnitude, further confirms the robustness of our conclusions.

### 5.3. Robustness and Heterogeneous

To ensure the robustness of our research findings, we first carried out three robustness tests: (1) the core independent variable was lagged by one period, and the peer academic performance in the previous period was used to explain the high school students’ academic performance in the current period; (2) the dependent variable and the core independent variable were replaced with the self-evaluated academic performance and its mean of the students; (3) due to the truncation feature of the achievement data, we replaced the estimation with the Tobit model.

The results of the robustness tests are shown in Table 10. Evidently, our conclusions exhibit robustness, and various models lend support to our conclusions to different extents.

In addition to the aforementioned robustness checks on reverse causality, alternative measurements, and model specifications, we conducted a placebo test to further investigate whether the model suffers from mechanical correlation or systematic bias. Specifically, we employed the student’s birth month as a falsification outcome. The underlying logic is that birth month is a quintessential predetermined trait that, theoretically, cannot be influenced by the current academic performance of peers. As reported in Model (17) of Table 11, the regression results show that the core independent variable is statistically insignificant (*p* = 0.233) in predicting birth month. This result provides no evidence that the model generates systematic associations with biologically predetermined traits, thereby reducing concerns about mechanical correlation or systematic bias and offering additional support for the internal coherence of the empirical strategy.

To ensure that the estimated peer effects are not sensitive to the choice of econometric specifications, we employ the control function approach as an alternative identification scheme, supplementing our primary 2SLS analysis. Beyond serving as a cross-method validation, the control function approach provides a more intuitive statistical diagnostic by explicitly incorporating the first-stage residuals. Following the identification strategy in Model (11), we utilize the first-lagged academic performance of second-order peers as the instrumental variable in this specification.

The results are presented in Model (18) of Table 12. The first-stage results demonstrate that this instrumental variable exhibits strong predictive power. The Wald test significantly rejects the null hypothesis of weak instruments, further reinforcing the validity of our network-based identification strategy. In the second-stage estimation, the coefficient of the first-stage residual term is statistically significant at the 0.1% level, which not only provides empirical justification for the necessity of our instrumental variable strategy but also reveals the underlying endogenous bias within the original estimates.

After correcting the standard errors using 1000 bootstrap resamples, the estimated coefficient for peer average performance stands at 1.121 (*p* < 0.001). This estimate is highly consistent with the 2SLS result (1.072) in terms of sign, magnitude, and statistical significance. The slight increase in the coefficient magnitude suggests that the control function approach may capture the endogenous nuisance parameters with greater precision. The high degree of convergence between these two distinct methodologies provides additional support for the stability of the results and suggests that the findings are not driven by environmental confounding or endogeneity concerns.

Due to the current rapid development in China, with the influence of economic and social systems, individual factors, and school factors, the sample may have heterogeneity. Therefore, we conducted further heterogeneity analysis on the results in Table 13. Models (19) and (20) were conducted from the perspective of household registration, and the peer effect of the sample from urban areas was significantly greater than that of the sample from rural areas. Models (21) and (22) were conducted from the perspective of gender, and the peer effect of the female sample was significantly greater than that of the male sample, which may be due to the fact that male students are more active, thereby widening the variability of their friends’ academic performance. Models (23) and (24) were conducted from the perspective of schools, and the peer effect of the sample from county schools was greater than that of the sample from town schools, which may be because county schools have better educational conditions and play a prominent role in improving the peer’s academic performance.

To further explore potential heterogeneity across different developmental stages, we introduced an interaction term between the peer mean score and student age. The results, as presented in Model (25) of Table 14, show that the estimated coefficient of the interaction term is 0.001 with a *p*-value of 0.923. This indicates that the interaction is statistically insignificant, suggesting that the magnitude of peer influence does not vary systematically with age. Consequently, the primary findings are robust across the age spectrum in our sample, demonstrating that the identified peer effect is consistent throughout the transition from early to late adolescence.

## 6. Discussion

This paper, based on the questionnaire and social network survey data of high school students in a typical county-level area in western China, and on the foundation of constructing a theoretical analysis framework of the peer effect on academic performance, empirically tests the peer effect on the academic performance of high school students. The results indicate that within this county-level context, peers with high academic performance are positively and significantly associated with the academic performance level of high school students. After overcoming the endogenous issues brought about by self-selection and reflection problem, the estimated peer effect remains statistically significant under the maintained identification assumptions. These findings provide contextually grounded evidence for county-level high school settings in western China. Given that the selected county is broadly representative in terms of its geographic location, level of socio-economic development, cultural context, and strong institutional emphasis on education, the conclusions of this study may reasonably extend to other western Chinese counties that share similar structural and educational environments.

This study examines the moderating mechanisms of peer influence from three dimensions: the overall demonstration effect of the peer network, the within-class network effect, and the average effect of cross-class peers. These findings not only verify how peer quality conditionally shapes the transmission of academic influence but also reveal the complex role played by social structure in this process.

First, the demonstration effect can be considered a primary pathway through which high-quality peers influence academic performance. Students change by observing, imitating, and internalizing their peers’ behavioral patterns and academic standards. When the quality of the peer network is high, students are more frequently exposed to visible and imitable models of success, thereby reinforcing their learning pathways. Empirically, the significantly positive mediated moderation effect, together with the significant negative direct effect, indicates that the demonstration mechanism is the dominant channel. The negative sign of the direct effect may reflect competitive pressure or upward social comparison in high-quality environments; however, this negative component is substantially offset by the strong mediated pathway, ultimately resulting in a significantly positive total effect. These patterns are consistent with the notion that high-quality peers are associated with improved academic outcomes through the cognitive and behavioral internalization generated by visible success models, rather than simple social proximity.

Second, the within-class network effect exhibits an unexpected asymmetric dependence logic. The interaction coefficient is negative, which differs from the conventional intuition that more social resources always lead to stronger spillover effects. This deviation reveals how students with different network positions and academic attributes absorb external information differently. For students situated at the periphery of the network and with limited direct access to resources, particularly those with lower academic standing, peer performance can act as an external reference for perceiving academic standards and acquiring advanced information. This relative sense of resource scarcity makes them highly sensitive to small fluctuations in peer performance, which is associated with larger marginal effects. In contrast, students occupying central network positions, with more stable social foundations and resource reserves, display stronger autonomy and social buffering capacity in their academic performance. The resource saturation associated with central network positions, combined with stronger academic preparedness, is associated with reduced reliance on external peer influence, which may contribute to greater academic stability amid group fluctuations, explaining why the marginal impact of peer influence diminishes as their network position and resources improve.

Third, the analysis of the cross-class average effect further expands the value of social boundaries. Although the cross-class moderation coefficient does not reach a highly significant statistical level, its positive sign provides directional evidence. Unlike the dense and highly homogeneous familiar networks within a class, cross-class interactions provide heterogeneous resource supplementation. Such ties can act as social bridges, potentially associated with access to non-redundant academic resources and diverse ways of thinking that are less available within the focal class. Although this influence appears more dispersed due to greater social distance, leading to lower statistical certainty, these patterns are consistent with a potential pathway for academic development, suggesting that external connections may offer informative value even if the effect is not statistically significant. These weak connections, though less frequent than within-class interactions, possess unique and irreplaceable value in breaking informational echo chambers and providing differentiated support.

## 7. Conclusions

This study provides systematic and credible evidence on the association between peer performance and high school students’ academic outcomes in a typical county-level context in western China. Building on extensive prior research, it makes several potential contributions. First, at the data and variable level, given the challenges of obtaining comprehensive social network information in real-world settings, we leveraged a census-style survey of typical county schools in China to construct a complete peer network encompassing 3847 students. Based on this network, we further constructed core variables, enabling a more precise and comprehensive quantification of peer effects. Compared with prior studies relying on sampled data, the completeness of both the dataset and variable design allows a more rigorous empirical investigation of academic peer effects from a social network perspective, yielding both theoretical and practical significance. Second, at the theoretical level, we offer a more nuanced understanding of the mechanisms underlying peer influence. Beyond examining the demonstration effect of the complete network, we separately investigate within-class network effects and cross-class average effects, thereby capturing multi-dimensional transmission pathways and enriching the conceptualization of peer influence. This multi-level framework provides a more systematic perspective on how peer interactions shape academic outcomes. Third, in addressing potential endogeneity, we adopt a layered identification strategy, combining rich individual controls, school-by-grade fixed effects, and class-level random intercepts. Together with instrumental variable techniques, control function approaches, and a series of robustness checks, our methods ensure the reliability and generalizability of the core findings while preserving the natural structure of the peer networks. This approach provides a rigorous and practical framework for addressing endogeneity and identifying peer effects in densely connected academic settings. In sum, this study contributes to the literature by integrating data and variable completeness, theoretical clarity, and methodological rigor, providing systematic and credible evidence on peer effects in high-density academic environments.

Certainly, this paper has some areas for improvement. Firstly, the research conclusions are derived from cross-sectional data from the 2023 baseline survey, providing a static snapshot of peer dynamics. To enhance the robustness of our interpretations regarding peer influence, future research should transition toward longitudinal designs that track the co-evolution of social networks and academic achievement over time. By leveraging future data, we plan to employ dynamic network modeling to better account for the endogenous nature of peer selection. This will allow exploration of how the formation, persistence, and dissolution of ties relate to student trajectories, while accounting for unobserved time-invariant individual characteristics that often confound peer effect estimates in cross-sectional settings.

Secondly, while this paper identifies the demonstration effect as a key mechanism, the behavioral pathways through which peer influence operates could be more granularly explored. Future research designs should aim to integrate more multidimensional data, such as detailed activity logs of peer interactions or records of collaborative learning tasks, to provide more concrete evidence of the social learning process. By matching social network data with these process-oriented metrics, subsequent studies can move beyond measuring performance outcomes to identifying the specific spillover of study habits and behavioral norms. This would allow for a more rigorous empirical test of how peer quality is internalized and translated into individual academic growth.

Thirdly, our evidence stems from a typical county in western China. While our findings provide valuable insights into peer dynamics within this specific educational context, their external validity across different institutional settings remains to be tested. Future studies could employ comparative frameworks to investigate whether the observed moderated mediation effects hold constant in regions with different educational resource allocations, school management models, or socio-economic backgrounds. Such research would help identify the boundary conditions of our theory and ensure its applicability to a broader range of educational environments.

Overall, the research findings of this paper provide actionable guidance for educational interventions in underdeveloped regions. By fostering high-quality peer interactions within classes, tailoring support according to students’ network positions, for example, providing additional guidance for peripheral students while leveraging central students as peer facilitators, and encouraging cross-class connections, educators and policymakers can enhance the flow of academic information, reduce disparities, and leverage peer-mediated learning. Such targeted strategies can help optimize learning opportunities and improve overall student performance in resource-constrained contexts.

## Figures and Tables

**Table 1:** Descriptive statistics of variables.

Variable	Mean	Std. Dev.	Min	Max
Academic performance	0.575	0.156	0	1
Weighted average score of within-class peers	0.487	0.263	0	1
Weighted average score of cross-class peers	0.190	0.233	0	1
Peer quality	0.503	0.361	0	1
Demonstration probability	0.538	0.299	0	0.999
Within-class moderator	0.174	0.150	0	0.647
Cross-class moderator	0.019	0.034	0	0.411
Age	16.198	0.933	14	19
Gender	0.530	0.499	0	1
Whether they live in school	0.985	0.122	0	1
Study effort	58.080	20.591	0	100
Educational expectation	7.176	0.870	1	8
Self-confidence	2.799	0.703	1	4
Academic performance requirements	3.074	0.780	1	4
Health	3.615	0.916	1	5
Household registration	0.098	0.297	0	1
Family structure	4.780	0.695	1	5
Family economic status	2.799	0.608	1	5
Parents’ emotional status	4.048	0.907	1	5
Number of children	1.787	0.478	1	4
Extracurricular education expenditure	0.042	0.145	0	1
School education expenditure	0.303	0.250	0	1
Other education expenditure	0.182	0.252	0	1
Family education time investment	0.021	0.094	0	1
Family collection of books	0.180	0.236	0	1
Whether family members could speak English	0.686	0.464	0	1
The number of friends	6.673	8.904	0	121
The degree of study effort of friends	2.351	0.589	1	3
The number of friends with bad behavior	1.193	0.448	1	3
The number of friends who had been class leaders	2.135	0.526	1	3

**Table 2:** Estimation results of peer effects on academic performance.

Variable	(1)	(2)	(3)	(4)	(5)
${\bar{y}}_{-i}$		0.128 *** (0.013)	0.103 *** (0.011)	0.088 *** (0.011)	0.090 *** (0.011)
Control variables			Yes	Yes	Yes
Exogenous effects				Yes	Yes
School-by-grade fixed effects					Yes
ICC	0.565	0.528	0.580	0.580	0.539
Observations	3847	3847	3847	3847	3847

Note: *** denotes significance level of 0.1%.

**Table 3:** The moderation effect of peer quality.

Variable	(6)
${\bar{y}}_{-i}$	0.354 *** (0.013)
$PQ_{i}$	−0.183 *** (0.004)
${\bar{y}}_{-i}\times PQ_{i}$	0.243 *** (0.023)
Control variables	Yes
Exogenous effects	Yes
School-by-grade fixed effects	Yes
ICC	0.498
Observations	3847

Note: *** denotes significance level of 0.1%.

**Table 4:** The mediated moderation role of demonstration probability.

Variable	(7)	(8)
	$DP_{i}$	Academic Performance
${\bar{y}}_{-i}$		0.466 *** (0.013)
$PQ_{i}$	0.741 *** (0.006)	−0.040 *** (0.007)
$DP_{i}$		−0.210 *** (0.009)
${\bar{y}}_{-i}\times DP_{i}$		0.622 *** (0.063)
${\bar{y}}_{-i}\times PQ_{i}$		−0.270 *** (0.058)
Control variables	Yes	Yes
Exogenous effects	Yes	Yes
School-by-grade fixed effects	Yes	Yes
ICC	0.019	0.441
Observations	3847	3847

Note: *** denotes significance level of 0.1%.

**Table 5:** The network effect at the within-class level.

Variable	(9)
${\bar{y}}_{-i,\mathrm{cls}}^{w}$	0.102 *** (0.015)
${\bar{y}}_{-i,\text{non-cls}}^{w}$	0.096 *** (0.013)
$M_{i}^{cls}$	0.041 * (0.016)
${\bar{y}}_{-i,\mathrm{cls}}^{w}\times M_{i}^{cls}$	−0.084 (0.051)
Control variables	Yes
Exogenous effects	Yes
School-by-grade fixed effects	Yes
ICC	0.538
Observations	3847

Note: *** and * denote significance levels of 0.1% and 5%, respectively.

**Table 6:** Marginal effects of within-class peer performance on academic achievement.

$M_{i}^{cls}$	Marginal Effect
−1 SD	0.186 *** (0.052)
Mean	0.102 *** (0.015)
+1 SD	0.018 (0.055)

Note: *** denotes significance level of 0.1%.

**Table 7:** The average effect at the cross-class level.

Variable	(10)
${\bar{y}}_{-i,\mathrm{cls}}^{w}$	0.090 *** (0.012)
${\bar{y}}_{-i,\text{non-cls}}^{w}$	0.085 *** (0.015)
$M_{i}^{non-cls}$	−0.024 (0.059)
${\bar{y}}_{-i,\text{non-cls}}^{w}\times M_{i}^{non-cls}$	0.225 (0.243)
Control variables	Yes
Exogenous effects	Yes
School-by-grade fixed effects	Yes
ICC	0.539
Observations	3847

Note: *** denotes significance level of 0.1%.

**Table 8:** Estimation results of instrumental variables.

Variable	(11)		(12)	
	Stage 1	Stage 2	Stage 1	Stage 2
${\bar{y}}_{-i}$		1.072 *** (0.064)		0.960 *** (0.101)
IV	0.440 *** (0.059)		0.046 *** (0.007)	
Cragg-Donald Wald F		952.936		184.826
Kleibergen-Paap rk Wald F		56.427		44.529
Kleibergen-Paap rk LM		33.888		27.139
Control variables	Yes	Yes	Yes	Yes
Exogenous effects	Yes	Yes	Yes	Yes
School-by-grade fixed effects	Yes	Yes	Yes	Yes
R-squared	0.507		0.407	
Observations	3648	3648	3648	3648

Note: *** denotes significance level of 0.1%.

**Table 9:** The regression estimation results of the long format data.

Variable	(13)	
	Stage 1	Stage 2
${\bar{y}}_{-i}$		0.600 *** (0.090)
IV	0.016 *** (0.002)	
Cragg-Donald Wald F		3037.989
Kleibergen-Paap rk Wald F		52.372
Kleibergen-Paap rk LM		36.570
Control variables	Yes	Yes
Exogenous effects	Yes	Yes
School-by-grade fixed effects	Yes	Yes
R-squared	0.362	
Observations	25,532	25,532

Note: *** denotes significance level of 0.1%.

**Table 10:** Robustness test of regression results.

Variable	(14)	(15)	(16)
	One-Phase Lag	Replace Variables	Replace Model
${\bar{y}}_{-i}$	0.144 *** (0.016)	0.234 *** (0.026)	0.355 *** (0.043)
Control variables	Yes	Yes	Yes
Exogenous effects	Yes	Yes	Yes
School-by-grade fixed effects	Yes	Yes	Yes
ICC	0.518	<0.001	
Observations	3749	3749	3847

Note: *** denotes significance level of 0.1%.

**Table 11:** Placebo test using birth month as the falsification outcome.

Variable	(17)
${\bar{y}}_{-i}$	0.464 (0.389)
Control variables	Yes
Exogenous effect	Yes
School-by-grade fixed effects	Yes
ICC	<0.001
Observations	3847

**Table 12:** Estimation results of the control function approach.

Variable	(18)	
	Stage 1	Stage 2
${\bar{y}}_{-i}$		1.121 *** (0.124)
IV	0.232 *** (0.013)	
CF residual of ${\bar{y}}_{-i}$		−0.856 *** (0.050)
Wald $\chi^{2}$	341.690	3037.989
Control variables	Yes	Yes
Exogenous effects	Yes	Yes
School-by-grade fixed effects	Yes	Yes
ICC	0.508	0.104
Observations	3648	3648

Note: *** denotes significance level of 0.1%.

**Table 13:** Analysis of sample heterogeneity.

Variable	(19)	(20)	(21)	(22)	(23)	(24)
	Household Registration		Gender		School	
	Urban	Rural	Boy	Girl	County	Town
${\bar{y}}_{-i}$	0.107 ** (0.036)	0.095 *** (0.012)	0.084 *** (0.017)	0.104 *** (0.016)	0.095 *** (0.012)	0.059 * (0.030)
Control variables	Yes	Yes	Yes	Yes	Yes	Yes
Exogenous effects	Yes	Yes	Yes	Yes	Yes	Yes
School-by-grade fixed effects	Yes	Yes	Yes	Yes	Yes	Yes
ICC	0.526	0.545	0.527	0.544	0.588	0.236
Observations	377	3470	1809	2038	3311	536

Note: ***, ** and * respectively denote significance levels of 0.1%, 1% and 5%.

**Table 14:** Testing for peer effects heterogeneity by age.

Variable	(25)
${\bar{y}}_{-i}$	0.089 *** (0.012)
Age	−0.014 *** (0.003)
${\bar{y}}_{-i}\times \mathrm{Age}$	0.001 (0.010)
Control variables	Yes
Exogenous effect	Yes
School-by-grade fixed effects	Yes
ICC	0.539
Observations	3847

Note: *** denotes significance level of 0.1%.

## Data Availability

The data presented in this study are available on request from the corresponding author due to privacy reasons.

## Supplementary Materials

The supporting information can be downloaded at: https://www.mdpi.com/article/10.3390/bs16030370/s1.

## Appendix A. Baseline Model and Its Decomposition

Since high school students’ peers are not only in the same class but may also belong to different classes or grades, we decompose the endogenous peer effect to serve the mechanism test. For a given student i, the peer set $N\left(i\right)$ can be divided into within-class peers $N_{\mathrm{cls}}(i)$ and cross-class peers $N_{\text{non-cls}}(i)$. The sum of peers’ scores can then be expressed as:

(A1) $$\sum_{j\in N(i)}y_{j}=\sum_{j\in N_{\mathrm{cls}}(i)}y_{j}+\sum_{j\in N_{\text{non-cls}}(i)}y_{j}.$$

We define the average scores of within-class and cross-class peers as:

(A2) $${\bar{y}}_{-i,\mathrm{cls}}=\frac{\sum_{j\in N_{\mathrm{cls}}(i)}y_{j}}{\left|N_{\mathrm{cls}}(i)\right|},\;{\bar{y}}_{-i,\text{non-cls}}=\frac{\sum_{j\in N_{\text{non-cls}}(i)}y_{j}}{\left|N_{\text{non-cls}}(i)\right|}.$$

Accordingly, the overall average peer score ${\bar{y}}_{-i}$ can be written as a weighted sum of these two components:

(A3) $${\bar{y}}_{-i}=w_{-i,\mathrm{cls}}\cdot {\bar{y}}_{-i,\mathrm{cls}}+w_{-i,\text{non-cls}}\cdot {\bar{y}}_{-i,\text{non-cls}},$$

where the weights are defined by the relative size of each peer subgroup:

(A4) $$w_{-i,\mathrm{cls}}=\frac{\left|N_{\mathrm{cls}}(i)\right|}{\left|N(i)\right|},\;w_{-i,\text{non-cls}}=\frac{\left|N_{\text{non-cls}}(i)\right|}{\left|N(i)\right|}.$$

Substituting Equation (A3) into the baseline model gives the decomposition formula of the overall peer effect:

(A5) $$y_{i}=\alpha +\rho w_{-i,\mathrm{cls}}{\bar{y}}_{-i,\mathrm{cls}}+\rho w_{-i,\text{non-cls}}{\bar{y}}_{-i,\text{non-cls}}+\beta {\bar{X}}_{-i}+\gamma X_{i}+\delta_{g(i)}+u_{c(i)}+\varepsilon_{i}.$$

Formally, the effect sizes of $w_{-i,\mathrm{cls}}{\bar{y}}_{-i,\mathrm{cls}}$ and $w_{-i,\text{non-cls}}{\bar{y}}_{-i,\text{non-cls}}$ are equal, as implied by the derivation above. In practice, however, due to statistical errors and separate parameter estimation, we only require that the parameters be approximately equivalent rather than exactly equal. Accordingly, Equation (A5) is adjusted as follows:

(A6) $$y_{i}=\alpha +\rho_{\mathrm{cls}}w_{-i,\mathrm{cls}}{\bar{y}}_{-i,\mathrm{cls}}+\rho_{\text{non-cls}}w_{-i,\text{non-cls}}{\bar{y}}_{-i,\text{non-cls}}+\beta {\bar{X}}_{-i}+\gamma X_{i}+\delta_{g(i)}+u_{c(i)}+\varepsilon_{i}.$$

This is the regression model used in the test of within-class and cross-class effect mechanisms.
