# Supplementary material for: Peer Effects on Academic Performance of High School Students in a County-Level Context of Western China: Empirical Evidence from Large-Scale Social Network Survey
Source: Behav Sci (Basel). 2026 Mar 5;16(3):370. doi: 10.3390/bs16030370 (PMC13024077; doi:10.3390/bs16030370)
Supplement: Supplementary file 1 [file behavsci-16-00370-s001.zip › behavsci-4157211-supplementary.pdf]

Supplementary Material

We include a rich set of control variables at the individual, family, and peer levels to reduce omitted variable bias.

(1) **Age**: measured in years.

(2) **Gender**: dummy variable, coded 1 if the child is female and 0 if male.

(3) **Whether they live in school**: dummy variable (1 = yes, 0 = no).

(4) **Study effort**: self-reported study effort.

(5) **Educational expectation**: ordinal scale (1 = drop out now, 2 = junior high, 3 = vocational school, 4 = vocational high school, 5 = general high school, 6 = junior college, 7 = bachelor, 8 = master or above).

(6) **Self-confidence**: ordinal scale (1 = no confidence at all to 4 = very confident).

(7) **Academic performance requirements**: parental expectation for child's academic ranking (1 = no specific requirement, 2 = class average, 3 = above average, 4 = top five in class).

(8) **Health**: self-rated health (1 = very poor to 5 = very good).

(9) **Household registration**: dummy variable, coded 1 for urban and 0 for rural.

(10) **Family structure**: categorical variable (1 = other relatives, 2 = single-parent, 3 = adoptive parents, 4 = stepfamily, 5 = both biological parents).

(11) **Family economic status**: subjective rating of family wealth (1 = very poor to 5 = very affluent).

(12) **Parents' emotional status**: ordinal scale (1 = very bad to 5 = very good).

(13) **Number of children**: discrete measure of sibling size (1 = one child, 2 = two, 3 = three, 4 = four).

(14) **Extracurricular education expenditure, school education expenditure, and other education expenditure**: continuous variables representing household education expenditures (all standardized to the 0-1 range).

(15) **Family education time investment**: hours per week spent on tutoring (all standardized to the 0-1 range).

(16) **Family collection of books**: count measure from survey (all standardized to the 0-1 range).

(17) **Whether family members could speak English**: dummy variable (1 = yes, 0 = no).

(18) **The number of friends**: statistical calculation from social networks.

(19) **The degree of study effort of friends**: ordinal measure of peers' study diligence (1 = none, 2 = one or two, 3 = all friends).

(20) **The number of friends with bad behavior**: ordinal measure of peers' deviant behavior (1 = none, 2 = one or two, 3 = all friends).

(21) **The number of friends who had been class leaders**: ordinal measure of peers' leadership roles (1 = none, 2 = one or two, 3 = all friends).
